# Genetic Diversity and Genome-Wide Association Study of Morphological and Quality Traits in Peach Using Two Spanish Peach Germplasm Collections

**DOI:** 10.3389/fpls.2022.854770

**Published:** 2022-03-21

**Authors:** Jorge Mas-Gómez, Celia M. Cantín, María Ángeles Moreno, Pedro J. Martínez-García

**Affiliations:** ^1^Department of Plant Breeding, Centre of Edaphology and Applied Biology of Segura, Spanish National Research Council (CEBAS-CSIC), Murcia, Spain; ^2^Department of Pomology, Experimental Station of Aula Dei-CSIC, Spanish National Research Council, Zaragoza, Spain; ^3^Department of Horticulture, Agrifood Research and Technology Centre of Aragon, Zaragoza, Spain

**Keywords:** peach, SNP, genetic diversity, sport, peach powdery mildew, trichomes

## Abstract

Peach [*Prunus persica* (L.) Batsch] is one of the most important stone fruits species in world production. Spanish peach production is currently the second largest in the world and the available cultivars in Spain includes a great source of genetic diversity with variability in fruit quality traits and postharvest disorders tolerance. In order to explore the genetic diversity and single nucleotide polymorphism (SNP)-trait associations in the Spanish germplasm, the new peach 18K SNP v2 array was used to genotype 287 accessions belonging to the two National Peach Germplasm Collections placed at the Agrifood Research and Technology Centre of Aragon (CITA) and at the Experimental Station of Aula Dei (EEAD)-CSIC. The high density of the new SNP array allowed the identification of 30 groups of synonymies, which had not been identified before using low-density markers. In addition, a possible large-scale molecular event in ‘Starcrest’, a sport of ‘Springcrest’, was detected showing a possible chromosome replacement of a 13.5 Mb region. Previous suggestions about Spanish diversification regions agreed with our genetic diversity and linkage disequilibrium (LD) decay results using high-density markers. A genome-wide association study (GWAS) detected 34 significant SNP-trait association with the type of leaf glands (TLG), fruit hairiness (FH), and flesh texture (FT). The impact of the significant SNPs was studied with SnpEff. Candidate genes encode several important family proteins involved in trichome formation and powdery mildew resistance (linked to TLG in peach). The genetic distance among cultivars obtained, together with SNP-trait associations found, provide new knowledge for marker-assisted selection and crossing approaches in peach breeding programmes.

## Introduction

Peach [*Prunus persica* (L.) Batsch] belongs to the Rosaceae family, genus *Prunus* (L.), and is the third most important temperate tree fruit in terms of worldwide production (FAOSTAT, 2021). Peach origin has been described in Asia and its domestication in China. From China, it was dispersed to Europe more than 2,000 years ago (Westwood, 1978; Hesse, 1985; Scorza and Sherman, 1996; Byrne et al., 2012). During the 16th century, peach was introduced to the Americas by the Spanish and Portuguese settlers, where after around 400 years the first formal breeding programme was established (Hesse, 1985; Scorza and Okie, 1990; Faust and Timon, 1995; Byrne et al., 2012).

Over the 1950s (1950–1960), researchers at the “Experimental Station of Aula Dei-CSIC” carried out an initial survey and collection aiming to determine and preserve the variability in the most cultivated stone fruit species that existed in Spain (Herrero et al., 1964). Thus, the first Spanish peach germplasm collection was established at the “Experimental Station of Aula Dei-CSIC” in Zaragoza (Spain), with the aim of organising, describing, and studying peach cultivars traditionally grown in Spain (Herrero et al., 1964). Initially, this collection was mostly composed of non-melting and clingstone cultivars due to their popularity among fruit growers and preferred consumption in Spain. In the successively propagated collections, during the following decades, peach cultivars with similar fruit typology were also introduced from other countries (Cambra, 1979a,b) aiming to study and select the most interesting accessions. In the case of the CITA collection, the exigency and interest of the European market for melting flesh cultivars, induced the establishment of a new peach collection focused on these types of fruit. From that moment on both collections worked coordinately, trying to include the maximum range of material used by Spanish growers. Germplasm banks are an efficient tool for genetic diversity preservation, providing phenotypic diversity to improve important traits in breeding. In fact, both collections studied here showed phenotypic variation for several important diseases and phenological and fruit quality traits (Font i Forcada et al., 2014; Cantín et al., 2021; Giménez et al., 2021).

The molecular analysis, of the different accessions maintained in the collections, allows the establishment of systematic relationships among accessions including their evolutionary relationships and the detection of misidentifications and possible errors made during other GeneBank operations (Bretting and Widrlechner, 1995). The Spanish peach germplasm has been previously explored using microsatellites or simple sequence repeats (SSRs), establishing genetic distances among accessions and marker-trait associations (Aranzana et al., 2003; Bouhadida et al., 2011; Alonso Segura et al., 2013; Font i Forcada et al., 2013). More recently, different resources using next-generation sequencing (NGS) technologies have been generated to study and manage peach germplasm collections (Verde et al., 2012, 2013, 2017), with the most recent being the new version of the peach 18K SNP v2 array (Gasic et al., 2019). Such tools are providing new knowledge for peach breeding regarding genetic control of fruit maturity (Pirona et al., 2013; Nuñez-Lillo et al., 2015; Elsadr et al., 2019), fruit quality and flower traits (Font i Forcada et al., 2019; Lobato et al., 2021), tolerance to diseases (Cirilli et al., 2017; Fu et al., 2021), agronomical traits (da Silva Linge et al., 2021; Fu et al., 2021), and genetic diversity (Thurow et al., 2020; Fu et al., 2021; Mas-Gómez et al., 2021). However, only a small fraction of the Spanish peach germplasm has been studied using NGS methodologies (Font i Forcada et al., 2019; Mas-Gómez et al., 2021).

More recently, the use of NGS methodologies has allowed the exploration of the somatic mutations occurring in bud sports in peach (Foster and Aranzana, 2018). In peach breeding, somatic mutations have been relatively frequent and used as a source of genetic variation (Okie, 1998; López-Girona et al., 2017; Foster and Aranzana, 2018). Such mutations can occur in specific histogenic layers (chimerical), generating individuals formed by genetically different cells (Burge et al., 2002; López-Girona et al., 2017; Foster and Aranzana, 2018). A chimerical natural mutation in the meristematic layer II revealed a new structural mutation causing the reversion from flat to round shape in peach (López-Girona et al., 2017). Moreover, specific regions with possible null alleles and different SNP genotypes in different groups of known sports have been identified using the previous 9K SNP v1 peach array (Micheletti et al., 2015).

Peach shows a high degree of self-compatibility which has caused considerable homozygosis levels in peach populations as the Spanish ones (Aranzana et al., 2003; Mas-Gómez et al., 2021), and probably helped to reduce genetic diversity (Li et al., 2013). In addition, a narrow genetic background has been found in peach breeding programmes due to the use of few founders (Li et al., 2013). Genetic diversity studies in peach have showed the highest level of genetic diversity in oriental peaches germplasm (Li et al., 2013), showing a fast decay of linkage disequilibrium (LD) in this population. However, peach germplasm of breeding programmes has shown similar patterns of LD and reduction of variability independently of the programme location (Xie et al., 2010; Micheletti et al., 2015). The higher number of alleles per locus of SSRs against the bi-allelic nature of SNPs, has frequently influenced genetic diversity and LD studies in peach, observing low values of genetic diversity and slow decays of LD when using SNPs (Micheletti et al., 2015; Thurow et al., 2020; Mas-Gómez et al., 2021). Population structure studies in peach have frequently shown differentiated groups by geographic origin (Font i Forcada et al., 2013; Li et al., 2013; Micheletti et al., 2015) and between melting and non-melting flesh varieties (Aranzana et al., 2010; Li et al., 2013; Chavez et al., 2014; Micheletti et al., 2015; Thurow et al., 2020). In Spanish peach germplasm, two diversification regions were identified by Bouhadida et al. (2011) suggesting the different regional environments and the peach industry requirements as main factors for the differentiation.

Here, we genetically characterised, on a genome-wide scale, the two National Peach Germplasm Collections placed at the Agrifood Research and Technology Centre of Aragon (CITA) and at the Experimental Station of Aula Dei (EEAD-CSIC), both located in Zaragoza (North Eastern Spain), and including most of the peach material traditionally used in Spain. We used the new peach 18K SNP v2 array to provide new findings of sports, synonymies/homonymies, SNP/trait associations of important fruit traits, and a deeper description of the population structure, genetic diversity, and LD decay.

## Materials and Methods

### Plant Material and DNA Extraction

A total of 287 accessions of *P. persica* (L.) Batsch from the CITA and EEAD-CSIC peach germplasm collections were studied. Both germplasm collections were established in different experimental field conditions at Zaragoza (in 2010 and 2005, respectively) (North Eastern Spain; latitude 41 43 42.7 N, longitude 0 48 44.1 W), and the trees were grafted onto the peach-almond hybrid ‘Garnem’ (CITA) and the hexaploid plum ‘Adesoto’ (EEAD-CSIC) rootstocks. Among the 287 accessions, 176 are Spanish, and 81 from the United States, whereas the rest come from Italy, France, South Africa, Russia, South America, and New Zealand. The Spanish accessions come from different regions located in the Ebro Valley and/or northeast Spain [Zaragoza (54), Lleida (47), Huesca (17), Navarra (8), Teruel (11), Bilbao (1), and La Rioja (1)] and three regions in southeast Spain [Murcia (24), Valencia (1), and Castellón (1)]. Moreover, 11 accessions from Spain whose region of origin is unknown were also studied. To assure and confirm genotyping results, replicates from 37 genotypes, located in both collections, were used. The set included peach and nectarines, round and flat fruit, and yellow or white flesh (see Supplexmentary Table 1 for details about the ID, origin, and fruit typology of the accessions). Genomic DNA was extracted from leaf tissue as described by Doyle and Doyle (1987). The samples were quality tested and quantified using a NanoDrop 2000 spectrophotometer (Thermo Fisher Scientific, Wilmington, DE, United States) and Qubit (Thermo Fisher Scientific, Wilmington, DE, United States), respectively.

### Genotyping

DNA samples were genotyped with the new version of the high-density Illumina peach 18K SNP v2 array (Gasic et al., 2019), using an iScan at the “Centre for Research in Agricultural Genomics” (CRAG) in Barcelona (Spain). Genotype calls for each SNP were obtained using the iScan output data in the Genotyping Analysis Module of GenomeStudio™ v2.0.5. (Illumina Inc., San Diego, CA, United States) using the default parameters. SNPs were filtered with the software ASSIsT v1.02 (Di Guardo et al., 2015) establishing a Frequency Rare Allele value of 0.05. SNPs classified as “Monomorphic”, “Failed”, and “NullAllele-Failed” were removed (Di Guardo et al., 2015). Subsequently, the SNPs which overcome the previous step with minor allele frequency (MAF) higher than 0.05, were filtered in the Genotyping Analysis Module of GenomeStudio™ v2.0.5 to be used as the high-quality subset of SNPs for further analysis (Vanderzande et al., 2019; Fu et al., 2021).

### Identification of Identical Genotypes and Labelling Errors

PLINK v.1.90 software (Purcell et al., 2007) was used to detect clones using genotype data to provide an identity-by-state (IBS) measure. Available known duplicates individuals were used to set the IBS threshold for clone detection. Input files used by PLINK were generated in GenomeStudio by “PLINK Input Report Plug-in v2.1.4” using the final subset of SNPs. The identical genotypes detected through PLINK were excluded for the subsequent analysis, with only one individual per group (with the higher Call Rate) retained. Also, four accessions of the complete set belonging to the CITA collection were excluded for subsequent analysis. All clone groups and homonymies detected here were verified when previous published data, using 9K SNP array, were available.

Parent–child (P-C) (between an individual and a single parent) and parent–parent–child (P-P-C) (combination of the two parents with the offspring) Mendelian-inconsistent errors were checked in those individuals with known pedigree information (Okie, 1998), to provide data and clarify possible uncertain cases (Vanderzande et al., 2019). In addition, P-P-C relationships determined as wrongs were re-analysed separating the parents to calculate P-C Mendelian inconsistent errors. Mendelian-inconsistent errors were obtained through GenomeStudio™ generating a “Reproducibility and Heritability” report. A threshold of 0.5% errors (Vanderzande et al., 2019) was established to consider a relationship as true.

### Genetic Diversity Analysis

Genetic diversity analysis was performed by measuring the fixation index (F_*ST*_), g_*ST*_, and D_*Jost*_ of the total accessions, and the allelic richness (A_*r*_), observed heterozygosity (H_*o*_), and expected heterozygosity (H_*e*_) of the populations. These data were obtained using the “basicStats” function of the DiveRsity v.1.9.90 package of R (Keenan et al., 2013). Moreover, the pairwise F_*ST*_, g_*ST*_, and D_*Jost*_ values among the identified populations were calculated using the “diffCalc” function. For these analyses, only the high-quality SNP subset was used, and only Spanish populations were analysed, excluding those with only one individual and those groups of clones with origin in more than one region (undetermined origin), unless historical data was available.

### Linkage Disequilibrium

Linkage disequilibrium was analysed using PLINK (Purcell et al., 2007). The *r*^2^ was calculated for each pair of SNPs in a maximum window of 100 subsequent SNPs or a distance of 5,000 kilobases (Kb). The *r*^2^ values were plotted against genetic distance using the *ggplot2* R package (Wickham, 2016). In addition, the set of SNPs was pruned for LD in PLINK using the command “–indep-pairwise.” The parameters were as follows: a window size of 50 SNPs, 5 SNPs to shift the window at each step, and an *r*^2^ threshold of 0.2. The LD analysis was carried out in the whole set of genotypes, and also in the subpopulations inferred by the genetic structure analysis.

### Genetic Structure Analysis

The pruned set of SNPs was used to perform the genetic structure analysis using fastStructure v.1.0 (Raj et al., 2014). Clusters (K) were set from 1 to 10. For the choice of the most likely K, “chooseK.py” script was used. In addition, a discriminant analysis of principal components (DAPC) was carried out in the R package *adegenet* v 2.1.3 (Jombart, 2008). DAPC optimises the variance between groups while minimises variation within clusters (Jombart and Collins, 2015). To identify the optimal number of clusters, the *k*-means algorithm was run and the solutions were compared using the Bayesian information criterion (BIC) to select the “best” number of clusters (Jombart and Collins, 2015). For the DAPC, it is required to retain a number of PCs, which can have a significant influence on the results. For this reason, the “cross-validation” procedure in two steps described by Jombart and Collins (2015) was used to obtain an optimal number of PCs. DAPC results were plotted using the ggplot v.3.3.3 R package (Wickham, 2016).

### Genome-Wide Association Study

Qualitative traits, such as flesh texture (FT) (melting/non-melting), fruit hairiness (FH) (hairy/glabrous), and the type of leaf gland (TLG) (globose/reniform) from the majority of the accessions used here were recorded for 3 years and were confirmed with historical records from previously published studies. FH was phenotyped by classifying fruit type from each tree as peach (hairy) or nectarine (glabrous) at harvest time. For the TLG attribute, at least 20 leaves from each tree were visually examined each year, following the UPOV guidelines for peach characterisation (UPOV, 2010). FT was phenotyped by sensory analysis by trained personnel, at consumption ripeness, by following the consensus definition for ‘Melting’ attribute: ‘ease with which the flesh disintegrates under a slight pressure exerted between the tongue and the palate’ (Harker et al., 2010). At least three fruits per tree were tasted (six fruits per accession) each harvest season. This information was used to perform a genome-wide association study (GWAS) analysis with the high quality SNPs to identify associations between SNPs and these important traits. This analysis was carried out in the GAPIT R package v.3.1. (Lipka et al., 2012), implementing a mixed linear model (MLM) using as input, the genotypic and phenotypic data, a kinship matrix (IBS), and population structure as cofactors (both generated through genotypic data by GAPIT). Significant associations between SNPs and traits were determined using a Bonferroni adjustment at the α = 0.05 level.

SnpEff v4.3e (Cingolani et al., 2012) was used, to predict the effects caused by the significant SNPs identified. Together with significant SNPs data, the peach reference genes annotations (v2.0.a1)^1^ were used as input. The SNP predicted effects were classified by impact: moderate (non-synonymous substitution), modifier (with impact on non-coding regions), low (synonymous substitution), or high (disruptive impact on the protein). Subsequently, the functional characterisation of each affected genes according SnpEff was carried using the gene list analysis tool from the PANTHER classification system (Mi et al., 2017). Finally, an overrepresentation test was carried out using a GO-SLIM annotation data set for each functional classification (molecular function, biological process, and cellular component), and all genes were listed in the peach genome, according to Fisher’s exact statistical test and Benjamini–Hochberg’s False Discovery Rate correction.

## Results

### Characterisation and Selection of Single Nucleotide Polymorphisms

A total of 16,038 SNPs were scored in GenomeStudio (no data was received from the remaining 1,962 SNPs). Six individuals with poor quality (Call Rate around 0.5–0.6) were excluded and the total of SNPs were re-clustered in GenomeStudio (Supplementary Table 1). ASSIsT determined 2,502 (15.6%) SNPs as monomorphic and 1,261 SNPs (7.87%) as failed (Table 1), and both groups were removed for downstream analysis (see Supplementary Table 2 for SNP classification done in ASSIsT). In addition, SNPs with a MAF lower than 0.05 were excluded. Eventually, a subset of 11,549 high quality SNPs was obtained (Table 1).

**TABLE 1:** SNP classification summary obtained in ASSIsT.

Category	Number of SNPs	% SNPs
Failed	1,122	7.00
Monomorphic	2,502	15.60
NullAllele-Failed	139	0.87
DistortedAndUnexpSegreg	5,246	32.71
OneHomozygRare_HWE	1,451	9.05
OneHomozygRare_NotHWE	2,127	13.26
Robust	1,274	7.94
ShiftedHomo	2,177	13.57
SNPs with MAF < 0.05 (passed in ASSIsT filtration)	726	4.53
High quality SNPs	11,549	72.01
Total	16,038	100.00

### Identification of Clones and Labelling Errors

A pairwise IBS analysis was carried out to detect identical genotypes (from now on called “clones”) and identify synonymies, homonymies, and sport mutations. The IBS threshold to detect clones was 0.99, using as reference the known sports of ‘Springcrest’ (‘Starcrest’, ‘Springbelle’, ‘Springlady’, ‘Maycrest’, and ‘Springold’) (see Supplementary Table 1 for clone and homonymies detection). Fifty-two groups of clones (containing 162 individuals) were detected, being group 7 (‘Calabacero’ individuals and ‘Selma’) and group 24 (‘Maruja’ individuals and ‘San Jaime’) the largest ones, with 8 individuals each. Only the individual with the highest call rate in each group was kept for the next analysis; thus, the set of studied individuals was reduced to 167. Among the replicates used in this study, 30 groups have been confirmed as true replicates, and seven have been detected as homonymies (‘Amarillo Calanda’, ‘Amarillo de Octubre’, ‘La Escola’, ‘Montañana’, ‘Paraguayo Almudí’, ‘Starn’, and ‘Vivian’). The replicate for ‘Baladín’ (‘Baladín V.T.’ from CITA) was not confirmed due to the low call rate of ‘Baladín’ accession. A double confirmation of the synonymies and homonymies detected here was carried out using previously available data. As result, misidentifications were detected in this germplasm and ‘Baby Gold-8 (2565)’ (actually ‘Baby Gold-6’), ‘Silver Rome (5414)’ (actually ‘Baby Gold-9’), ‘Shasta (2286)’ (actually ‘Fortuna’), ‘Tasty Free (5187)’ (actually ‘Rojo de Azagra’) were confirmed as clear labelling errors. In the case of ‘Vivian_2289’, ‘Merriam (5117)’, and ‘Croc Abel (3698)’ their genotypic data were different from the available data at GDR.

Another issue was detected with ‘Blanco Tardío’, collected from two different locations from the CITA collection (‘Blanco Tardío_J_5373’ and ‘Blanco Tardío_I_5373’). The two reps of ‘Blanco Tardío_I’ were identical and, according to PLINK, clones of ‘Fantasia’ (Clone Group 4). The sample ‘Blanco Tardío_J_5373’ was different from the two reps of ‘Blanco Tardío_I_5373’ and from ‘Fantasia’, which could indicate also a clear labelling error. Although this issue was not previously observed by the gene bank curators, it has now been checked and confirmed *in situ* by the authors.

Pedigree verifications in GenomeStudio showed only 4 out of 12 P-C and three out of six P-P-C verifications as true (Supplementary Table 3). The P-P-C relationship analysis showed that ‘Vivian_5206’ (from CITA) (Error = 0.09%) was the real accession for its homonym ‘Vivian_2289’ (from EEAD-CSIC) (Error = 50.42%) (Supplementary Table 3) and confirmed ‘Baladin’ as a self-pollination of ‘Vivian_5206’. Moreover, such analysis confirmed ‘Dixon’ and ‘Wiser’ as parents of ‘Klamt’, and ‘Walgant’ as a self-pollination of ‘Kakamas’.

Our results have also confirmed ‘Carson’ as the parent of ‘Adriática’ and ‘Tebana’, being both genotypes synonymies. In addition, our results have also shown that ‘Fortuna’ is not a parent of ‘Adriática’. P-C relationship among ‘Kakamas’ and ‘Oom Sarell_5136’, ‘Merriam’, and ‘Everts’, and all the relationships of ‘Lovell’ were detected as wrong. Lastly, ‘Late Legrand’ was confirmed as parent of ‘Summergrand’ and ‘Autumn Grand’, and ‘Andross’ as child of ‘Fortuna’.

### Genetic Diversity Analysis

After excluding clones and individuals with undetermined origin, a genetic diversity analysis was carried out with six Spanish populations ranging from 5 to 23 individuals (Table 2) and containing a total of 68 individuals. The largest populations, Lleida and Zaragoza, showed the highest values of A_*r*_ (1.665 and 1.646, respectively), while Navarra, the smaller one, showed the lowest value (1.405). H_*e*_ showed a similar pattern, being the H_*e*_ values in Zaragoza and Lleida, 0.255 and 0.248, respectively, and 0.175 in Navarra. Regarding H_*o*_, Murcia showed the highest value (0.245), and Navarra the lowest one (0.113). Inbreeding coefficient determined Zaragoza as the population with the highest value (0.322), while Murcia showed a negative value (−0.108), causing a decrease in the average value (0.203) (Table 2).

**TABLE 2:** Population genetic diversity statistics allelic richness (A_*r*_), observed heterozygosity (H_*o*_), expected heterozygosity (H_*e*_), and inbreeding coefficient (F_*IS*_) calculated by DiveRsity package.

Population	Size	A_*r*_	H_*o*_	H_*e*_	F_*IS*_
Zaragoza	23	1.646	0.158	0.255	0.322
Murcia	5	1.540	0.249	0.216	−0.108
Navarra	5	1.405	0.113	0.175	0.306
Lleida	21	1.665	0.199	0.248	0.145
Huesca	6	1.587	0.158	0.241	0.292
Teruel	8	1.495	0.126	0.198	0.260
Average	11	1.556	0.167	0.222	0.203

**FIGURE 1:**
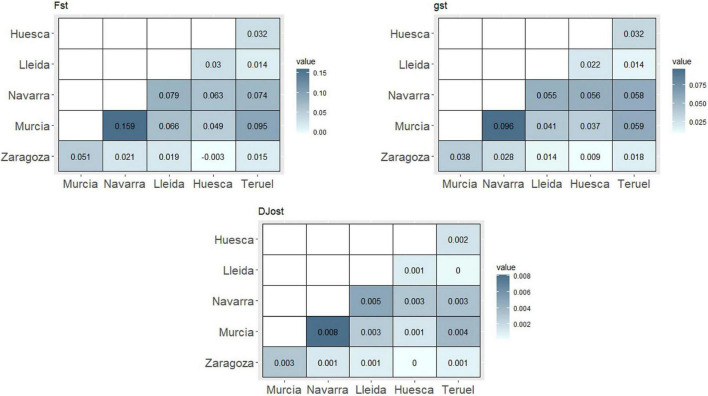
Genetic differentiation pairwise analysis among Spanish regions.

In general, pairwise analysis of genetic population differentiation showed a considerable distinction between population of Murcia (South of Spain) and the rest of the populations (North of Spain), although also Navarra (North of Spain) showed a moderate differentiation with other populations from others northern regions (Lleida, Huesca, and Teruel) (Figure 1). The highest values of F_*ST*_ were obtained between Murcia and the rest of the populations, being Murcia and Navarra (F_*ST*_ = 0.159) the more differentiated ones (Figure 1). Most of the north pairs showed low differentiation values, being Zaragoza and Huesca the most similar pairwise. The parameter g_*ST*_ showed the same pattern, reconfirming the highest differentiation between Murcia and Navarra (0.096) and the lowest ones between northern populations (Zaragoza and Huesca, 0.009). D_*Jost*_ values reconfirmed Murcia-Navarra as the more differentiated pairwise (0.008) and, on the other side, the pairwises Huesca–Zaragoza and Lleida–Teruel obtained a value of 0.

Global genetic values for the complete subset of Spanish accessions (without clones and individuals with unknown origin) were calculated (Supplementary Table 4). The F_*ST*_ value of 0.0326 indicated a low genetic differentiation among the populations, being 3.26% of the genetic variability related to interpopulation differences. D_*Jost*_ and g_*ST*_ values showed the same level of differentiation (0.006 and 0.0611, respectively). Global inbreeding values, F_*IT*_ and F_*IS*_ (0.3431 and 0.3209, respectively), indicated a considerable inbreeding in the whole population.

### Genetic Structure Analysis

Previously to the genetic structure analysis, the set of SNPs was pruned, using 167 individuals in the pruning process, achieving a final dataset of 612 reliable SNPs. First, the genetic structure was studied with fastStructure. The results of the *chooseK.py* script showed *K* = 6 as which maximises marginal likelihood (Marginal Likelihood = −0.9470914152) and it was selected to describe the population structure (Supplementary Figure 1).

**FIGURE 2:**
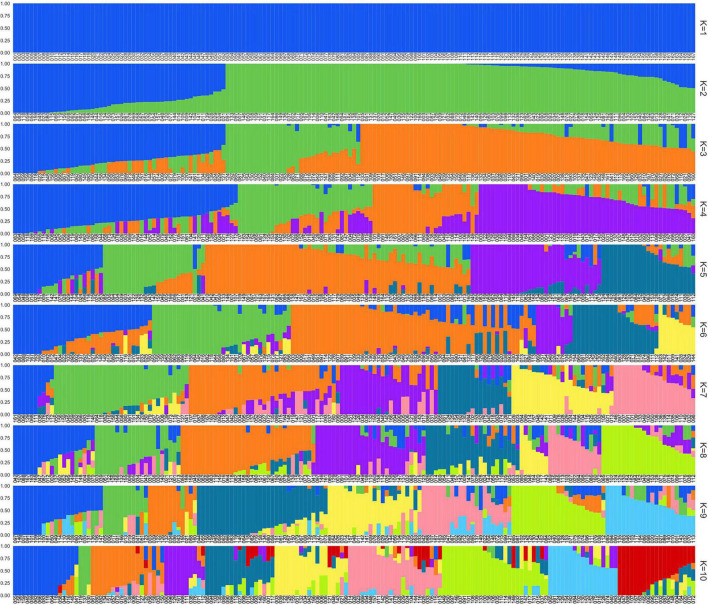
Population structure analysis results from *k* = 1 to *k* = 10. ID is described in Supplementary Table 1.

Considering a membership threshold of 0.75, the six clusters comprised 91 individuals and ranged from 2 (Cluster 6) to 33 (Cluster 3) (Figure 2). Seventy-six of the accessions showed a membership value lower than 0.75, indicating a considerable admixture in different clusters (Figure 2 and Supplementary Table 1). Cluster 1 is formed mostly by yellow-fleshed and non-melting peaches, all of them from the north of Spain. The vast majority of accessions in cluster 2 are melting flesh nectarines with origin in the United States. Cluster 3 is entirely formed by non-melting flesh peaches, except for ‘Pavía Blanca’ which is a non-melting nectarine. Moreover, in cluster 3, all the accessions are from Spain, including most of the accessions with origin in the south (‘Clone Group 20’, ‘Campillo Rocho’, ‘Jerónimo’, and ‘Jerónimo Balate’). Cluster 4 is formed by seven accessions, including mostly non-melting flesh peaches. The origin of accessions from cluster 4 is the United States, Brazil, Spain, and two unknown origins. Cluster 5 is fully formed by peaches, mostly non-melting fleshed and with origin in the United States, except for the clone group 19 with unknown origin, which includes the accessions ‘Jerónimo Espuña’ from Murcia and ‘Loadel’ from the United States. Cluster 6 includes two accessions, a melting flesh flat peach from Zaragoza (‘Paraguayo San Mateo’) and a melting flesh peach from Russia (‘Pace 03-14’). The rest of the flat peaches showed a high affinity for this cluster (values between 0.54 and 0.63) except for ‘Paraguayo Francia’.

The DAPC was carried out using values from *K* = 1 to *K* = 20, retaining the maximum number of PCs feasible (around 170 PCs) (Supplementary Figure 2). The BIC Values graph (Supplementary Figure 3) showed good values of *K* from *K* = 6 to *K* = 10, and finally *K* = 6 was selected.

**FIGURE 3:**
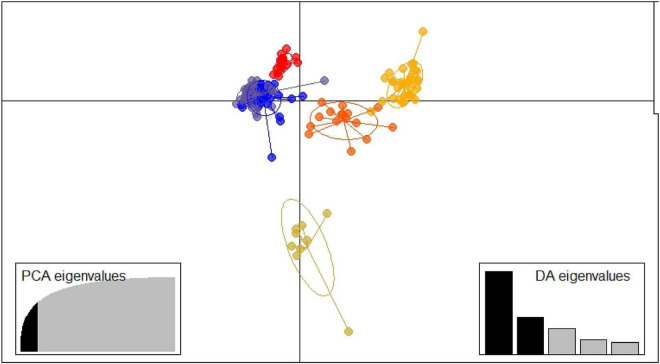
Discriminant analysis of principal components structure plot *k* = 6. ID is described in Supplementary Table 1.

The function *xvalDapc* indicated 19 as the number of PCs with the lowest root mean squared error (Supplementary Figures 4, 5). Finally, together with the 19 PCs, five discriminants functions were saved, and the conserved proportion of the variance was 0.656. The two main axes of the discriminant analysis were used to draw a scatterplot for the representation of the six clusters (Figure 3).

The six clusters determined by the DAPC ranged from 9 accessions (Cluster 6) to 47 (Cluster 1) (Supplementary Table 1). Cluster 1 comprised mostly yellow and white fleshed peaches from the north of Spain, including all the accessions from Navarra, the accessions ‘Nuevo’, ‘Starn_5488’ and ‘Garau’ from the United States, and ‘Chucho Picudo’ from Chile. Cluster 2 contains the majority of the melting flesh accessions and nearly all the studied nectarines in this work (Supplementary Figures 6, 7). Moreover, 24 of the 32 individuals of the cluster come from the United States (Supplementary Figure 8). Cluster 3 includes accessions mainly from the north of Spain and most of the accessions from the south of Spain, South Africa, and New Zealand (Supplementary Figure 8). All members of this cluster are non-melting yellow flesh peaches except for the white peach from Huesca ‘Pigat Susagna’ (Supplementary Figure 9). Cluster 4 includes non-melting peaches only from the United States, except for the clone group 19 with undetermined origin. Cluster 5 contains accessions from different origins (North of Spain, United States, Italy, and Brazil) being mainly non-melting flesh peaches. Cluster 6 comprises the majority of the flat peaches of the study, the Russian cultivars (‘Pace 03-14’ and ‘Pace 03-13_Paimet Simerenco’) and two non-melting flesh peaches [‘Comodin’ and ‘Montaced (Binaced)’].

The population genetic structure analysis through two different approaches allows the observation of some agreement between them. Supplementary Figure 10 shows the agreement between the clustering in fastStructure and in DAPC, only including the 91 individuals not admixed in fastStructure. A total agreement between the two approaches in the clusters FAST2/DAPC2, FAST4/DAPC5, FAST5/DAPC4, and FAST6/DAPC6 can be observed. The cluster FAST3 coincides with the cluster DAPC3 and the cluster FAST1 with the cluster DAPC1, except for the accession ‘Pavía Blanca’.

### Linkage Disequilibrium

The LD was studied in the final set of 167 individuals, using the high quality 11,549 SNPs. The *r*^2^ values were calculated and plotted according to the genetic distance of the SNPs pairwise (Supplementary Figure 11). The averaged *r*^2^ value in the whole set was 0.26, and overall, *r*^2^ was lower than 0.2 at around 1,500 Kb. For Cluster 4 and Cluster 6, LD decay was not estimated. LD decay varied for the rest of cluster, and in general, a low decay of LD was observed; overall, *r*^2^ was lower than 0.2 around 3,000 Kb (data not shown).

### Genome-Wide Association

**FIGURE 4:**
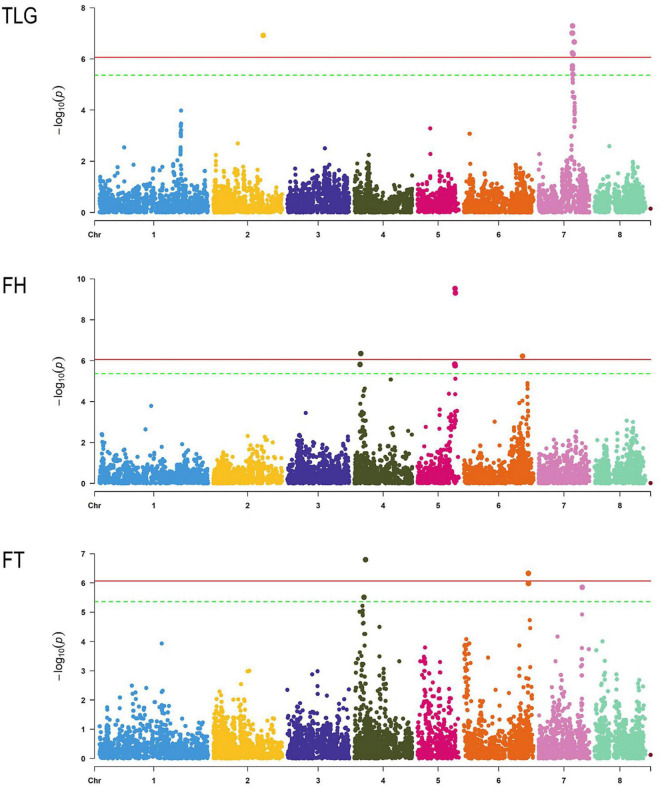
Manhattan plot of each trait studied through GWAS. Chromosomes are represented in the *x*-axis, the horizontal lines indicate the significance threshold with Bonferroni correction adjusted with α = 0.01 (red) and α = 0.05 (green).

Phenotypic data from 151 to 157 genotypes (depending on the trait) out of 167 of the total set was obtained. Regarding the TLG, 118 individuals had reniform and 34 globose glands. In the case of FH, 137 accessions were hairy and 20 were glabrous (Supplementary Table 5). Regarding FT, 31 genotypes were melting and 120 non-melting flesh. The threshold considering the Bonferroni correction to determine an SNP as significant was 4.33E−06. The analysis revealed a total of 34 SNPs associated with the traits (Figure 4 and Supplementary Table 6). We identified 15 SNPs with a strong association to FH, 9 of them placed in chromosome 5 in a region of 0.23 Mb (from 16,508,401 to 16,745,447 bp), including the two most significant SNPs-trait associations (Peach_AO_0589900 and Peach_AO_0590235). The rest of significant SNP associations to FH were found in chromosome 6 (Peach_AO_0677784) and in a region of 0.38 Mb in chromosome 4 (from 2,519,322 to 2,900,231 bp). Six SNPs with a strong association to FT were detected, two placed in chromosome 4 (SNP_IGA_386778 and SNP_IGA_389796), three in chromosome 6 covering a region of 0.01 Mb (from 28,456,008 to 28,466,341 bp) and one in chromosome 7 (SNP_IGA_786984). Finally, thirteen SNP associations to TLG were detected, identifying 11 SNPs in chromosome 7 covering a region of 0.9 Mb (from 14,753,057 to 15,679,702 bp) and two SNPs in chromosome 2 (SNP_IGA_275049 and Peach_AO_0288030).

The 34 significant SNPs detected were analysed using SnpEff, indicating 90 effects over 66 genes (Supplementary Tables 7, 8). The impact was low for 11 effects, moderate for 10, and modifier for 69. Most of the effects were detected in downstream (30 effects), in upstream (26 effects), and in exons (21 effects). A functional characterisation was obtained for 63 affected genes, out of 66, by Pather. The functional category domain with the highest number of hits was “Biological Process” with 46 hits, followed by “Cellular component” and “Molecular function” with 29 and 22 hits, respectively. Among the “Biological Process” terms, cellular process (GO:0009987) and metabolic process (GO:0008152) showed the highest number of hits (20 and 17, respectively), followed by cellular anatomical entity (GO:0110165) (16 hits) in the “Cellular component” category, and catalytic activity (GO:0003824) (13 hits) in the “Molecular function” domain. No statistically significant over- or under-represented terms were found in the enrichment overrepresentation analysis for this set of affected genes.

## Discussion

The wide genetic characterisation performed using the new peach 18K SNP v2 array has shown multiple synonymies and homonymies in the Spanish peach collections not detected previously with low-density markers (Bouhadida et al., 2011; Alonso Segura et al., 2013; Font i Forcada et al., 2013). The majority of detected synonymies and homonymies are in agreement with available genotypes in GDR. Nevertheless, misidentifications and labelling errors were detected. This kind of errors occurs frequently in germplasm collections because of mix up of accessions, errors in the entry of plants, or unavailability of accurate genomic tools to distinguish accessions (Reed, 2004; Vanderzande et al., 2019).

The different parameters, such as IBS and IBD, and specific thresholds (e.g., 0.97, 0.977, or 0.98), have been established previously to classify two individuals as identical using NGS (Micheletti et al., 2015; Vanderzande et al., 2019; Montanari et al., 2020). Here, the used IBS threshold of 0.99, based on the group of known sports of ‘Springcrest’ (Micheletti et al., 2015), has clearly helped to detect a large number of synonymies and homonymies, therefore, avoiding genetic errors and redundancies in the downstream analysis. In addition, an error threshold of 0.5% was used in pedigree verifications showing a considerable number of pedigree errors. The presence of validated and historical pedigrees (Okie, 1998) agreed with the conclusions made using GDR genotype data, per example showing ‘Fortuna’ as the parent of ‘Andross’ or showing mistakes in ‘Merriam’ as a self-pollination of ‘Everts_5058’.

According to the pairwise IBS analysis performed in PLINK, ‘Starcrest’ showed considerable genotype differences with the rest of the components of the ‘Springcrest’ sport group (clone group 35). These differences were detected in a region of 13.5 Mb located in the upper part of chromosome 4, where ‘Starcrest’ showed 139 SNPs with a different genotype and 362 SNPs with null alleles (being located between the heterozygous and homozygous clusters in GenomeStudio™) (Supplementary Figure 12). In addition, ‘Starcrest’ showed a reduction in heterozygous SNPs in such a region, in which only 93 out of 1,108 high quality SNPs were heterozygous. Previously, eight SNPs with different genotypes and a large amount of possible null alleles were detected in the same region by Micheletti et al. (2015) between ‘Starcrest’ and ‘Springtime’, another sport of ‘Springcrest’ not present in these collections. These findings may indicate the existence of a chimeric mutation in the upper part of ‘Starcrest’ chromosome 4. The chromosome replacement of the detected region by the homologous would explain the high homozygosity found, as it has been previously suggested for other peach sports (López-Girona et al., 2017; Aranzana, 2021). Moreover, the SNP placement between heterozygous and homozygous clusters observed in the SNP plots, may indicate the existence of some cell layer(s) with heterozygous genotype(s) (Supplementary Figure 12). Previous studies have found SNPs associated with the harvest date in the upper part of chromosome 4 (Font i Forcada et al., 2019; da Silva Linge et al., 2021). This fact could be related with the observed harvest date of ‘Starcrest’, being earliest than the rest of their sports (Supplementary Tables 9, 10).

Despite of the commented precision of the new SNP array to distinguish between ‘Starcrest’ and ‘Springcrest’, two inconsistencies were generated by the SNPs, since accessions with differences in flesh colour (yellow and white) were considered as clones [clone group 22 (‘Sudanell’ group) and 31 (‘Rojo de Tudela’–‘Tambarría’)]. In this sense, the yellow flesh is caused by the accumulation of carotenoids in the chromoplasts due to the disruption of the allele ccd4 in LG1, which prevents the degradation of carotenoids (Adami et al., 2013; Falchi et al., 2013). Three mechanisms of disruption of this allele have been described (Falchi et al., 2013) and the physical position of this gene was found from 25,639,600 to 26,317,783 bps (Adami et al., 2013). The 32 SNPs of the region comprised between 25,545,000 and 26,317,783 bps were checked manually being identical among the mentioned clone groups. Moreover, all SNPs found, in the previous study reported by Micheletti et al. (2015), placed in the array showed the same genotypes among clones here. A possible hypothesis of these two discrepancies could be the existence of a different (fourth) mechanism involved in flesh colour, which is not represented in the SNP array used here.

A general low H_*o*_ mean was observed in this study which can be explained by the smaller size of populations in comparison with other studies (Aranzana et al., 2010; Chavez et al., 2014; Micheletti et al., 2015; Thurow et al., 2020), except in the case of Murcia population where H_*o*_ was higher than H_*e*_, showing negative F_*IS*_ values (F_*IS*_ = −0.108). High rates of selfing in the northern Spanish populations could be the main reason of these results, showing highest F_*IS*_ values. In addition, this fact could also explain the slow decay of the LD observed here. All these results are clear descriptors of traditional Spanish accessions, which were selected from seed-propagated populations and selfing was the main mating system (Badenes et al., 1998; Aranzana et al., 2003).

According to previous studies (Herrero et al., 1964; Bouhadida et al., 2011; Cornille et al., 2015), the low and moderate differentiation values observed here, can be explained by a close origin of the used accessions and intense spread of cultivars along Spanish regions with similar growing conditions by fruit growers. At the same time, moderate values of differentiation were observed among Navarra population and some other relatively close northern populations, suggesting a different genetic background. The moderate differentiation of Murcia population with the rest of the populations, probably could be explained by the large geographical distance and their different soil and climatic conditions, which was also commented by Bouhadida et al. (2011).

As previously commented, the degree of population structure influences LD patterns within the genome (Thurow et al., 2020). In our study, clear differences were observed in the decay of LD between individual clusters, being slow in general. This was mainly due to the small sample size of each cluster. In this sense, a more rapid decay of LD was observed considering the whole population. High overall levels in the whole set have been observed in other species as wheat (Chao et al., 2010) with clear variation of LD decay among clusters. Based on this fact, a population level analysis was not considered in our study. In general, population structure analysis, by fastStructure and DAPC, divided the germplasm into well-defined clusters according to their genetic structure and fruit characteristics (flesh texture and typology).

Similar results have been previously observed (Aranzana et al., 2003, 2010; Chavez et al., 2014; Thurow et al., 2020). Interestingly, North American cultivars were mainly grouped in two clusters, which can be explained by their close relationships (Supplementary Figure 8; Okie, 1998; Li et al., 2013). A detailed understanding of the germplasm structure and clusters helps in parental selection in breeding programmes, increasing genetic diversity, and improving the potential gain from the selection (Pandey et al., 2021). In this sense, as preliminary results (after 1 year of phenotyping) some tolerant cultivars to *M. fructicola* were observed such as ‘Fraga B.D.’ and ‘Gallur’ (Cantín et al., 2021), which are related to FAST3, while ‘Montaced’, also tolerant (Cantín et al., 2021), had a higher affinity for FAST6. Regarding chilling injury susceptibility, the most tolerant cultivars, also according to preliminary results (Giménez et al., 2021) are distributed in FAST1 (‘Josepet’, ‘Plácido’, ‘Zaragozano Rojo’, ‘Risol’, and ‘Bonet IV’), FAST2 (‘Rubi Rich’), FAST3 (‘Zaragozano Amarillo’, ‘Oom Sarell’, ‘Baladín’, and ‘Alcañiz’) and FAST5 (‘Andross’, ‘Carson’, ‘Loadel’, and ‘Adriática’). These results provide new choices to breeders in parental selection and crossing designs.

The association analysis showed several strong SNP-trait associations with the studied traits. The leaf-gland phenotype trait acquired relevance because of glands contribution in biologic control (Mathews et al., 2009). In addition, a clear association of absence of leaf-glands with high susceptibility to peach powdery mildew (PPM) has been suggested (Dirlewanger et al., 1996). This morphological trait has been used by breeders as a tool to select descendants with glands in peach breeding programmes (Lambert et al., 2020). This trait was described as a Mendelian trait being the glands absence (ee), globose (Ee), and reniform (EE) (Connors, 1922) and placed on chromosome 7 (Dettori et al., 2001; Micheletti et al., 2015). More recently, using NGS technologies, Lambert et al. (2020) demonstrated that a MITE-like Moshan transposable element inserted in the candidate gene (*Prupe.7G121100*; 14,436,305–14,437,630 bp) controlling this trait, was responsible for the absence or globose-shape phenotype. SNP_IGA_776653 was the closest SNP to the candidate gene detected by Lambert et al. (2020), placed 0.3 Mb of the gene.

More interestingly, SnpEff analysis showed modifier effects in gene *Prupe_7G125700*, such gen encoded an ankyrine repeat domain (ANK) protein. This protein family has been associated to plant immunity response against different pathogens (Cao et al., 1997; Yan et al., 2002; Yang et al., 2012; Vo et al., 2015). In fact, a recent study identified a *MELO3C002434* candidate gene for resistance to powdery mildew in melon, which encodes an ANK protein (Cao et al., 2021). Moreover, several KASP markers were designed around this gene obtaining successful results for marker-assisted breeding (MAS) in melon (Cao et al., 2021). Therefore, *Prupe_7G125700* could play an important role in PPM resistance being a candidate gene for further studies in peach.

In the case of FH, seven new SNPs associated with this trait in this work were detected close to the retrotransposon insertion (from 15,897,836 to 15,899,002 bp) on chromosome 5 associated previously with the pubescence (Vendramin et al., 2014). These results agree with previous studies, showing that the region identified here could be an important conserved haplotype in glabrous individuals (Micheletti et al., 2015; Cao et al., 2016; Thurow et al., 2020; Tan et al., 2021). In fact, two significant SNPs (SNP_IGA_602331 and SNP_IGA_602512) located in this region and detected here, using mainly Spanish germplasm, were also detected with strong associations to FH by Micheletti et al. (2015). More recently, Tan et al. (2021) detected the highest associated SNP with FH in the same region (chr5:16,633,286; G/A), located ∼700 kb downstream of the major gene controlling this trait (MYB gene Prupe.5G196100). Although, these authors commented that no proper candidate genes are located in this region (Tan et al., 2021). The use of SnpEff in our analysis allowed us to detect the effect of the significant SNPs in the region in several candidate genes (*Prupe_5G208400*, *Prupe_5G208500*, *Prupe_5G210500*, and *Prupe_5G208100*). These genes belonging to important gene families involved in trichome formation and development in other species (Alvarez-Buylla et al., 2000; Li et al., 2011). Trichomes are hair-like appendages caused by the differentiation of epidermal cells (Vendramin et al., 2014). Modifier effects were detected in the genes *Prupe_5G208400* and *Prupe_5G208500*, orthologous with MADS-BOX transcription factor genes. MADS-box proteins have been associated to trichomes development in *Arabidopsis* (Alvarez-Buylla et al., 2000; Willmann and Poethig, 2011), cotton (Lightfoot et al., 2008; Li et al., 2011), and petunia (Ferrario et al., 2004). In fact, a homology was detected between *Prupe_5G208500* and *AGL8* (AGAMOUS-LIKE 8), a MADS-box gene protein which showed high similarity with *GhMADS11*, a gene expressed specifically in cotton fibres (trichomes) and associated to fibre cell elongation (Li et al., 2011). Two modifier effects were detected in *Prupe_5G210500*, a gen orthologous with a bHLH transcription factor domain. Previous studies have identified bHLH TFs involved in trichome formation in *Arabidopsis* (Morohashi et al., 2007; Zhao et al., 2008) and tea plant (Liu et al., 2021). Specifically, the induction of two bHLH TFs, GL3, and EGL3, has been associated with triggering the trichome initiation pathways and the regulation of trichome development in a framework involving also MYB TFs (Morohashi et al., 2007; Zhao et al., 2008; Hao et al., 2021; Shangguan et al., 2021). In addition, modifier effects were detected with a gen orthologous with COBRA-LIKE 4 protein gene (*Prupe_5G208100*). COBRA-LIKE family genes were associated with important roles in specific types of cell expansion and cell wall biosynthesis, finding the COBRA-LIKE 9 highly expressed in trichomes (Jones et al., 2006; Brady et al., 2007). The detection of modifier effects in the mentioned candidate genes caused by significant SNPs detected here and two additional independent mutation events (in total three) in the major gene controlling this trait (Vendramin et al., 2014; Tan et al., 2021), may suggest a higher complexity in the expression of FH beyond from the proposed until so far.

Finally, we identified SNPs with significant association to flesh texture (FT) at the upper part of chromosome 4, in a region at the end part of chromosome 6 and in chromosome 7. FT has been related with endopolygalacturonase activity, and several previous studies have indicated the end region of chromosome 4 as the main region on the genetic control of peach texture (Peace et al., 2005; Meneses et al., 2016; Serra et al., 2017; Carrasco-Valenzuela et al., 2019; Giné-Bordonaba et al., 2020). Strong associated SNPs were identified in such region (Martínez-García et al., 2013; Micheletti et al., 2015; Thurow et al., 2020). In addition, SNPs strongly associated to this trait were also identified on chromosome 5 by several authors (Serra et al., 2017; Ciacciulli et al., 2018; da Silva Linge et al., 2021) and chromosome 8 (Ciacciulli et al., 2018). Previous significant SNPs detected by earlier studies using the 9K SNP array v1 (Micheletti et al., 2015; da Silva Linge et al., 2021) were included in our study, except one (SNP_IGA_821894 on chromosome 8). However, no significant association of these SNPs with FT was obtained here. A possible reason for this result may be the low number of melting accessions studied here (31 accessions), compared to the number of non-melting genotypes (120 accessions). This clearly affects the detection of the QTL for FT at the end part of chromosome 4 in our study, where the genetic control of FT has been previously identified, using a BC1 population (Dettori et al., 2001). Although the detected QTL in Chr4 could be a low probability event because of linkage mapping which uses few meiotic events.

On the other hand, a SNP detected here with high association with FT was SNP_IGA_477159 (*p*-value = 4.74E−04), located at 19,820,974 bp on scaffold 4. The SNP location is closed to the position of the two SNPs with strong association to flesh texture identified by Thurow et al. (2020), and located at 19,904,250 and 19,904,264 bp positions. These results also reflected the complex genetic architecture underlying this trait, which was also confirmed here due to the detection of significant SNPs in different regions or chromosomes (Peace et al., 2005; Martínez-García et al., 2013). Additional studies, including the development of more accurate ways of phenotyping this complex trait, must be developed to complete the genetic dissection of this central quality trait in peach.

The large number of synonymies and labelling errors detected, revealed the importance of genotyping germplasm collections with high-density markers to avoid redundancies and achieve an efficient management. Also, genomic rearrangements detected in ‘Starcrest’ (a sport of ‘Springcrest’) modifying regions related to harvest date indicate new lines of research. Genetic diversity results have shed new light in the predominant mating system in traditional Spanish peach varieties, the two main diversification regions (North and South of Spain) and the spread of peach cultivars. GWAS carried out detected new SNPs associated to TLG, FH, and FT using Spanish germplasm not previously analysed. The SnpEff analysis allowed the detection of PPM resistance and trichome development candidate genes to be further explored in breeding programmes. Finally, the genetic distance between accessions observed here, together with the phenotypic variation observed after evaluations of these materials, could ensure the adequate and representative diversity for future use in the improvement of important traits in peach breeding.

## Data Availability Statement

The original contributions presented in the study are included in the article/Supplementary Material, further inquiries can be directed to the corresponding author.

## Author Contributions

PJM-G, CC, and MM: conceptualization. PJM-G: methodology. JM-G, CC, MM, and PJM-G: investigation and writing – review and editing. PJM-G, CC, and JM-G: data curation and writing – original draft preparation. All authors contributed to the article and approved the submitted version.

## Conflict of Interest

The authors declare that the research was conducted in the absence of any commercial or financial relationships that could be construed as a potential conflict of interest.

## Publisher’s Note

All claims expressed in this article are solely those of the authors and do not necessarily represent those of their affiliated organizations, or those of the publisher, the editors and the reviewers. Any product that may be evaluated in this article, or claim that may be made by its manufacturer, is not guaranteed or endorsed by the publisher.

## Footnotes

1
https://www.rosaceae.org/species/prunus_persica/genome_v2.0.a1
