# Supplementary material for: Genetic Diversity and Genome-Wide Association Study of Morphological and Quality Traits in Peach Using Two Spanish Peach Germplasm Collections
Source: Front Plant Sci. 2022 Mar 21;13:854770. doi: 10.3389/fpls.2022.854770 (PMC8979248; doi:10.3389/fpls.2022.854770)
Supplement: Supplementary file 1 [file Data_Sheet_1.zip › Supplementary Figures.docx]

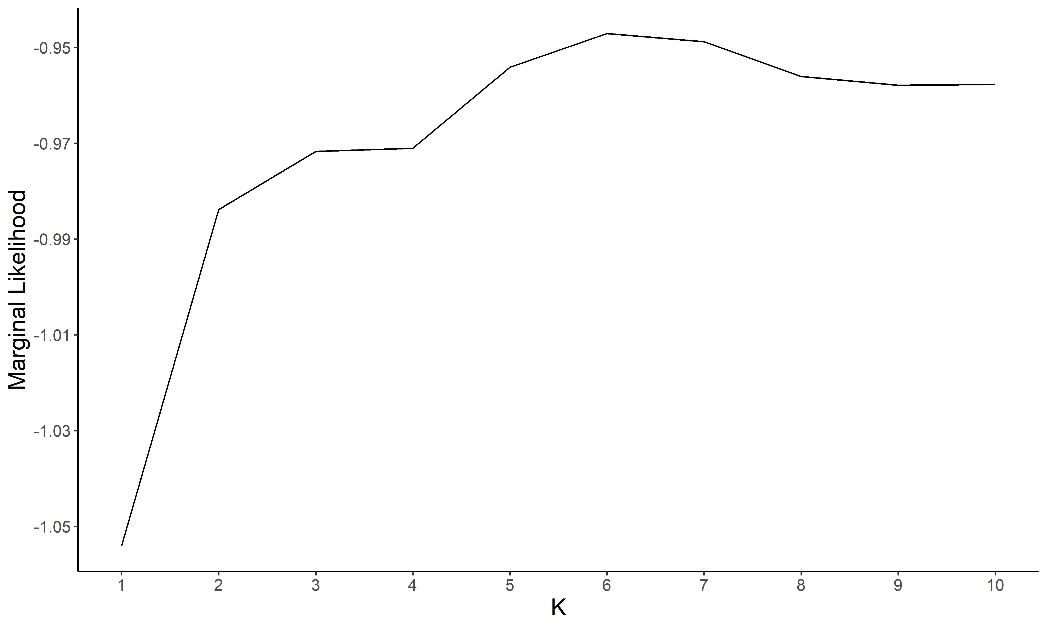


**Supplementary Figure 1.** Marginal likelihood per number of clusters obtained in fastStructure.


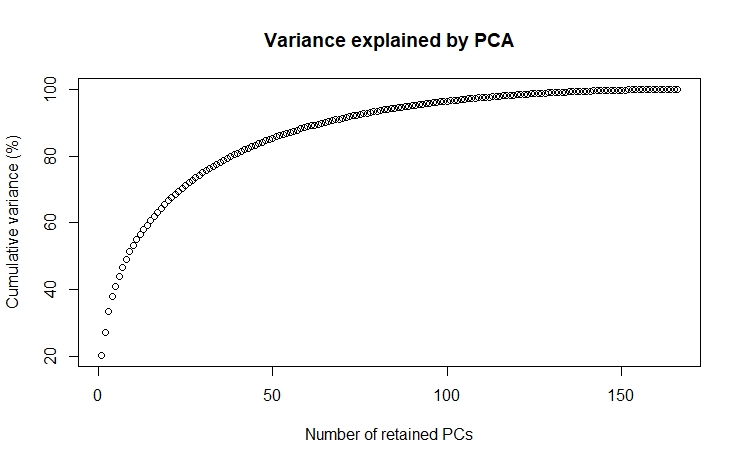


**Supplementary Figure 2.** Cumulative variance explained by PCA retained by PCs.


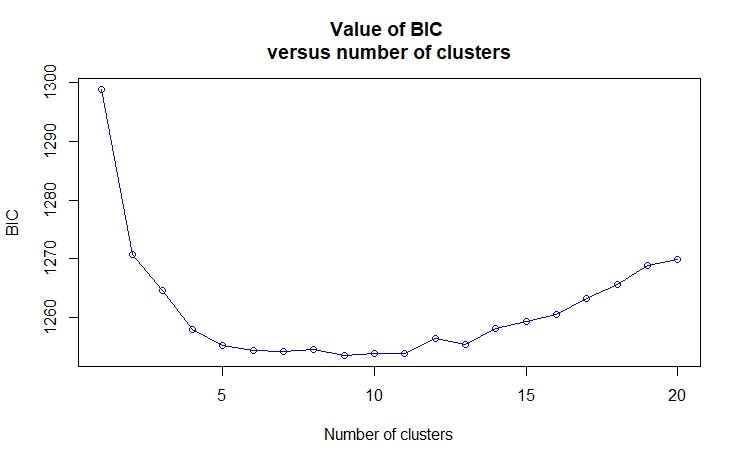


**Supplementary Figure 3.** Bayesian information criterion versus number of clusters.


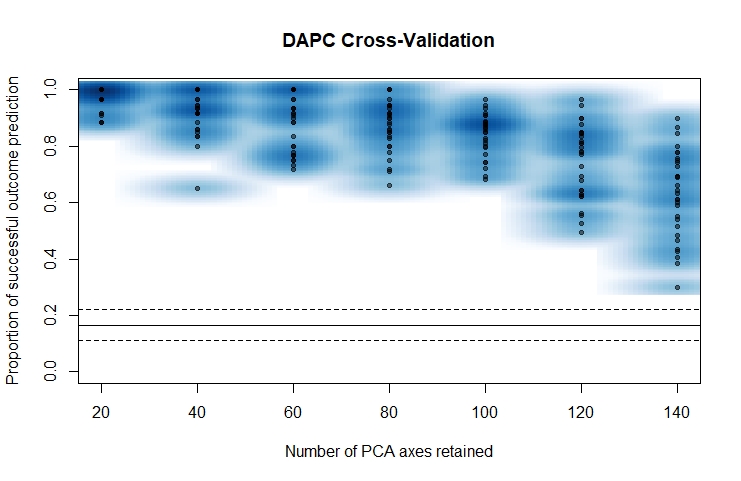


**Supplementary Figure 4.** DAPC Cross-Validation plot between 20 to 140 axes retained.


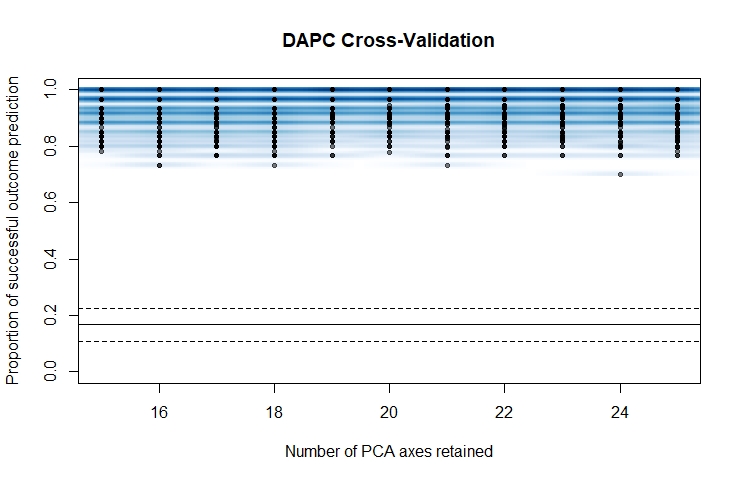


**Supplementary Figure 5.** DAPC Cross-Validation plot between 15 to 25 axes retained.


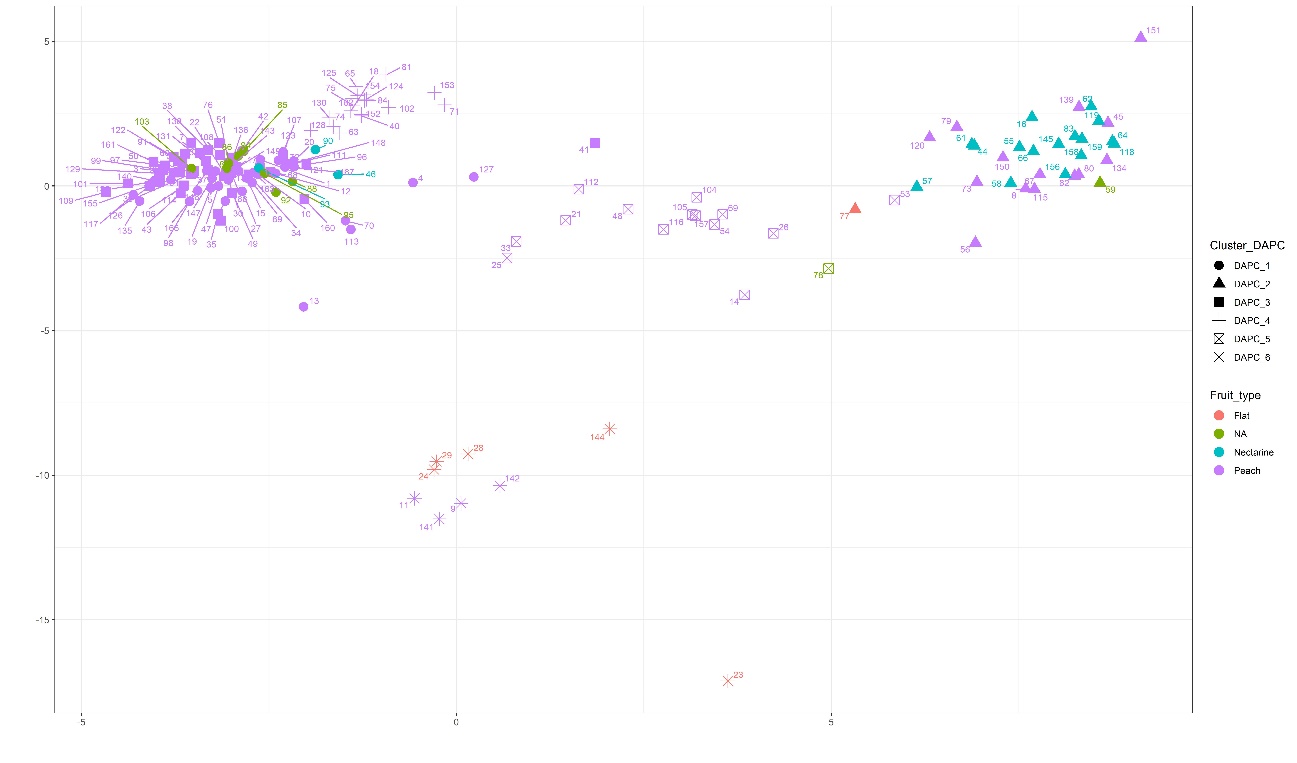


**Supplementary Figure 6.** DAPC cluster plot indicating fruit typology.


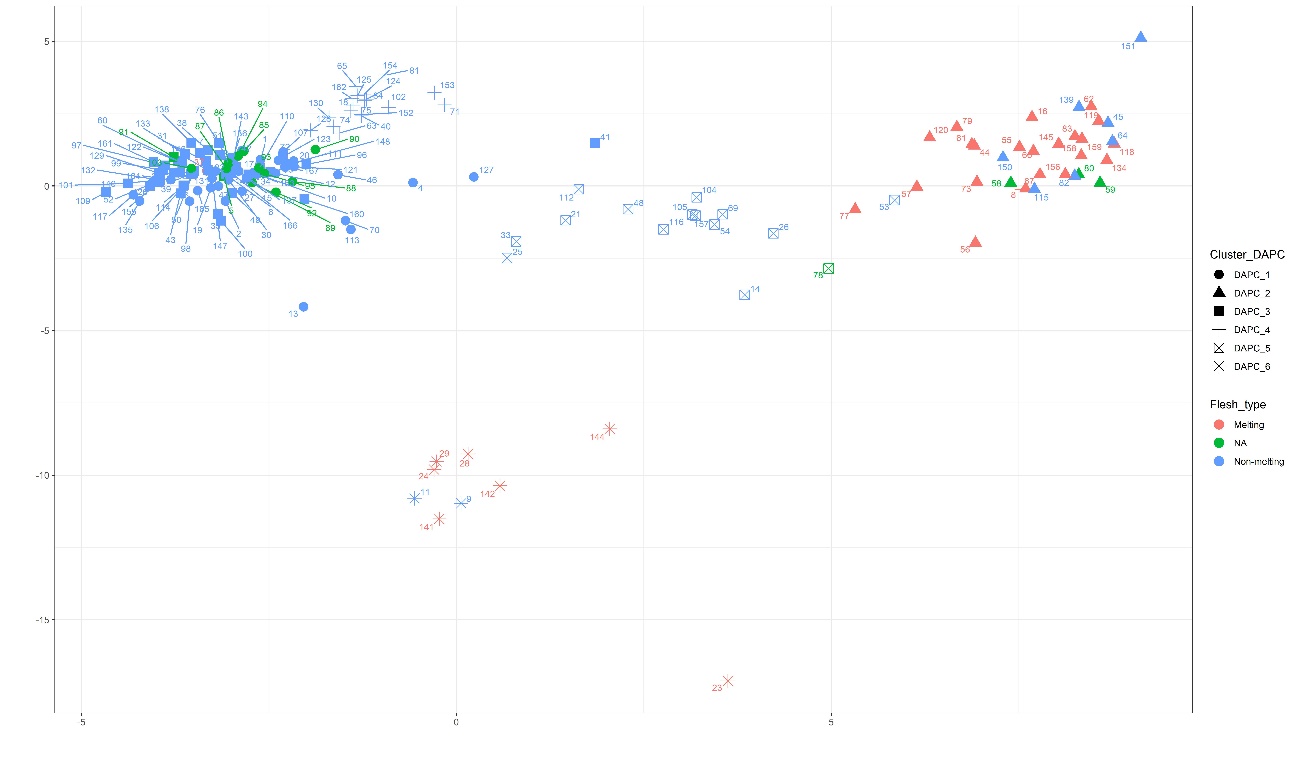


**Supplementary Figure 7.** DAPC cluster plot indicating fruit texture.


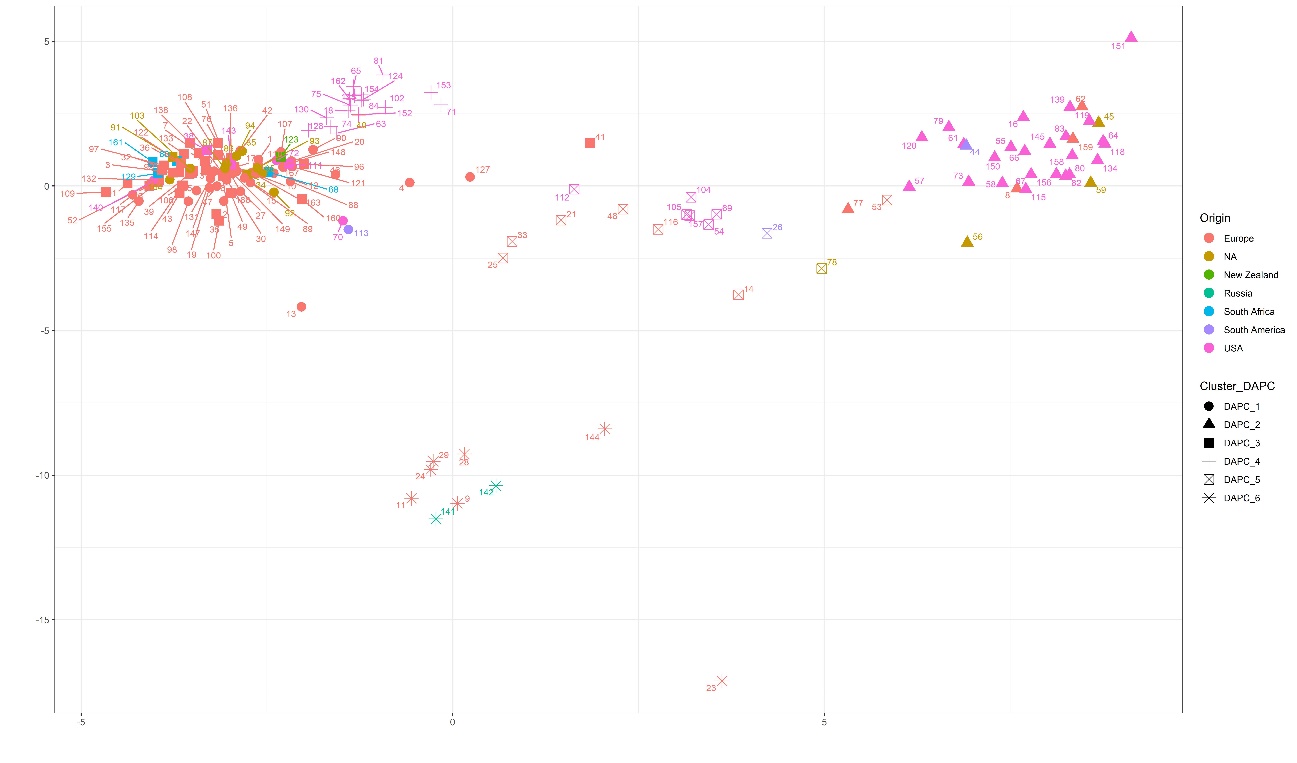


**Supplementary Figure 8.** DAPC cluster plot indicating geographic origin of the accessions.


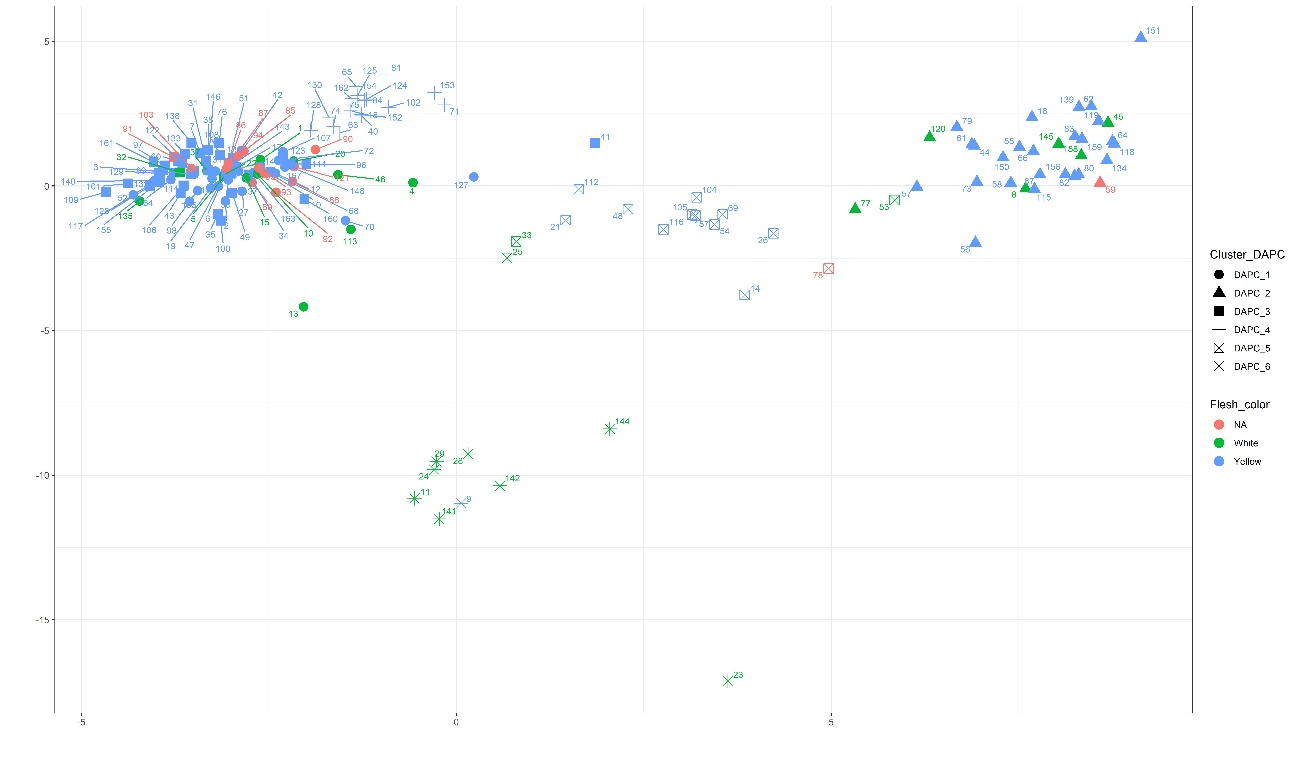


**Supplementary Figure 9.** DAPC cluster plot indicating flesh colour of the accessions.


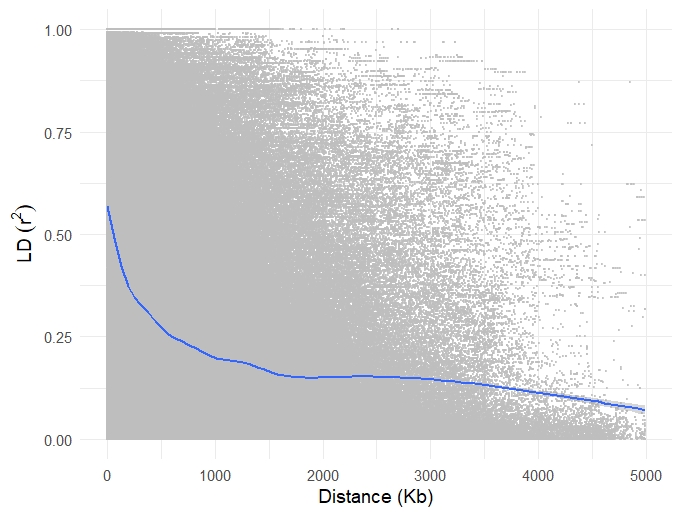

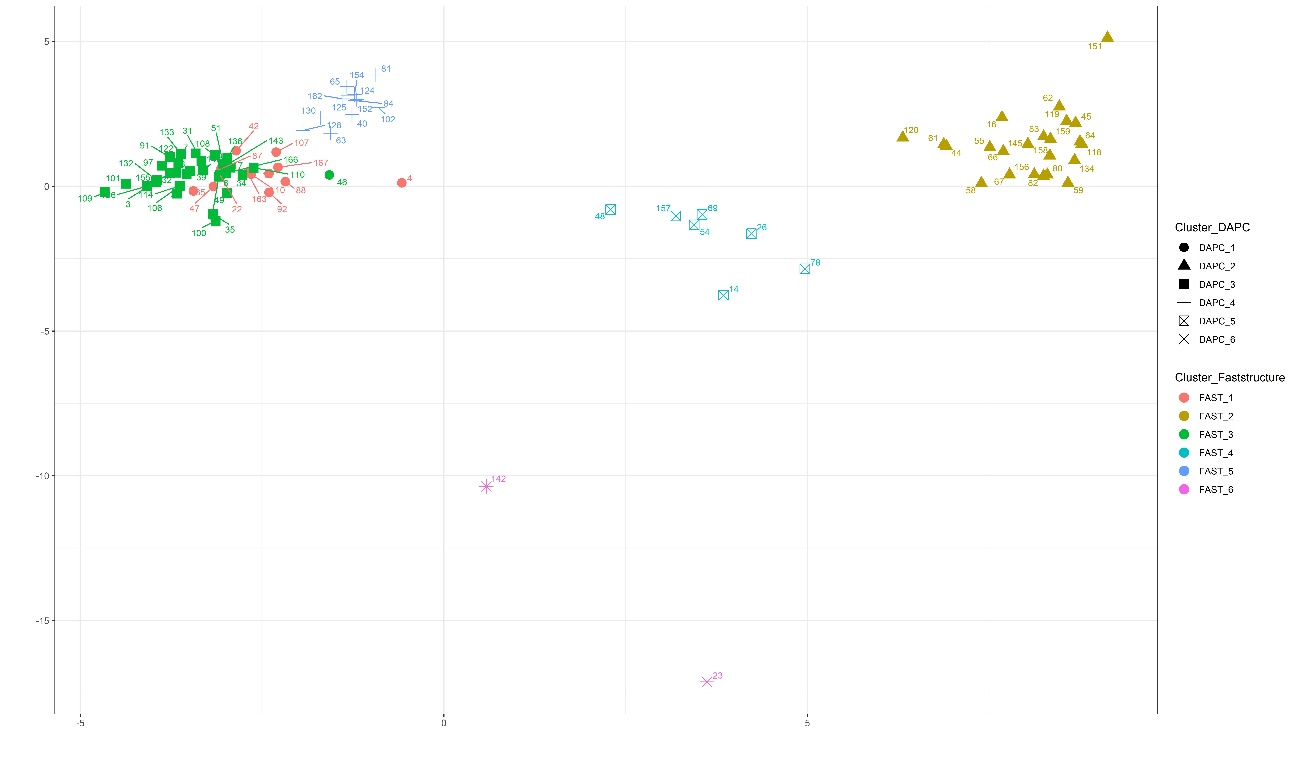


**Supplementary Figure 11.** Decay of linkage disequilibrium in the 167 individuals. Dots represent r^2^ values calculated.

**Supplementary Figure 10.** DAPC cluster plot showing agreement between fastStructure analysis and DAPC.


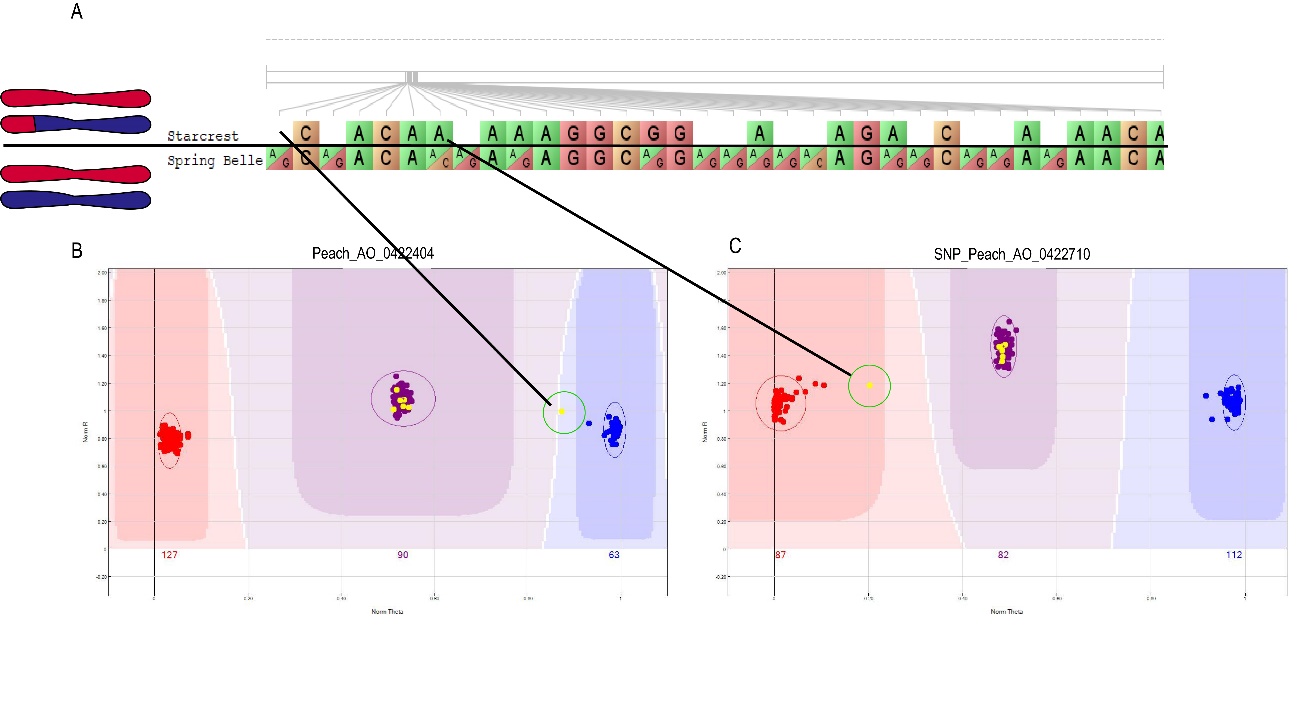


**Supplementary Figure 12.** Representation of chromosome 4 of `Starcrest´ and `Spring Belle´(as example) indicating their different genotypes using FlapJack software (a). SNP plots of two SNPs of the upper part of chromosome 4, highlighting in yellow the group of sports and encircling in green `Starcrest ´. Two SNPs are represented as example (b) a missing data SNP of `Starcrest´ and (c) a call SNP but separated of the homozygous BB cluster.
